# Genome-Scale Meta-analysis of Host Responses to *Staphylococcus aureus* Identifies Pathways for Host-Directed Therapeutic Targeting

**DOI:** 10.1093/infdis/jiaf290

**Published:** 2025-05-31

**Authors:** Clark D Russell, Seraphima Goeldner-Thompson, Emilie Smith, Jonathan E Millar, Bo Wang, Nicholas Parkinson, Sara Clohisey Hendry, Maaike Swets, J Ross Fitzgerald, J Kenneth Baillie, David H Dockrell

**Affiliations:** University of Edinburgh Centre for Inflammation Research, Institute for Regeneration and Repair, Edinburgh, United Kingdom; Baillie-Gifford Pandemic Science Hub, Institute for Regeneration and Repair, University of Edinburgh, Edinburgh, United Kingdom; University of Edinburgh Centre for Inflammation Research, Institute for Regeneration and Repair, Edinburgh, United Kingdom; University of Edinburgh Centre for Inflammation Research, Institute for Regeneration and Repair, Edinburgh, United Kingdom; Baillie-Gifford Pandemic Science Hub, Institute for Regeneration and Repair, University of Edinburgh, Edinburgh, United Kingdom; Roslin Institute, University of Edinburgh, Edinburgh, United Kingdom; Baillie-Gifford Pandemic Science Hub, Institute for Regeneration and Repair, University of Edinburgh, Edinburgh, United Kingdom; Roslin Institute, University of Edinburgh, Edinburgh, United Kingdom; Roslin Institute, University of Edinburgh, Edinburgh, United Kingdom; Baillie-Gifford Pandemic Science Hub, Institute for Regeneration and Repair, University of Edinburgh, Edinburgh, United Kingdom; Roslin Institute, University of Edinburgh, Edinburgh, United Kingdom; Roslin Institute, University of Edinburgh, Edinburgh, United Kingdom; Department of Infectious Diseases, Leiden University Medical Center, Leiden University, Leiden, The Netherlands; Roslin Institute, University of Edinburgh, Edinburgh, United Kingdom; University of Edinburgh Centre for Inflammation Research, Institute for Regeneration and Repair, Edinburgh, United Kingdom; Baillie-Gifford Pandemic Science Hub, Institute for Regeneration and Repair, University of Edinburgh, Edinburgh, United Kingdom; Roslin Institute, University of Edinburgh, Edinburgh, United Kingdom; University of Edinburgh Centre for Inflammation Research, Institute for Regeneration and Repair, Edinburgh, United Kingdom

**Keywords:** host-directed therapy, phagocyte, platelet, innate immunity, *Staphylococcus aureus*

## Abstract

**Background:**

*Staphylococcus aureus* infections are frequently complicated by metastatic foci, recurrence, and death. Antimicrobial resistance and intracellular bacterial persistence limit the effectiveness of conventional antimicrobials. Host-directed therapies could improve outcomes, but the interpretive complexity of pathogen–host interactions impedes identification of critical responses suitable for therapeutic targeting. To address this, we performed a meta-analysis of genome-scale studies aiming to prioritize host responses to *S aureus.*

**Methods:**

Lists of genes associated with host responses to *S aureus* were retrieved from systematically identified genome-scale studies, then integrated using the meta-analysis by information content (MAIC) algorithm. This generated a single aggregated gene list, ranked based on the cumulative evidence supporting each gene.

**Results:**

MAIC prioritized 3867 host genes. Myeloid cell immune responses were enriched with specific hubs including TLR2, IL-17, IFN-γ, and IL-1β. Noncanonical effector pathways were also enriched: autophagy (specific factors including mTOR and LAMP2), apoptosis (including BAD and BID), ferroptosis and iron metabolism (TFRC ranked 8/3876), and proteasomal antimicrobial responses (including PSME3 and the novel antimicrobial peptide PPP1CB). Prioritized genes were associated with genome-wide association study traits related to platelet count. In a cohort of patients with *S aureus* bacteremia, platelet count was differentially associated with clinical outcomes. Targets with immediate therapeutic relevance included *S aureus*/fibrin/platelet microthrombus formation (VWF, GP11b), *S aureus*–induced platelet loss (ASGR2), autophagy (mTOR), BID-mediated apoptosis, and intracellular bacterial killing (IFN-γ).

**Conclusions:**

This in silico analysis identifies cytokine hubs associated with the response to *S aureus* infection and prioritizes additional host responses including apoptosis, autophagy, iron metabolism, and thrombosis as therapeutic targets.

## Teaser

Integration of genome-scale data on host responses to *Staphylococcus aureus* identified the genes and pathways with the most cumulative evidence. This defined cytokine hubs and prioritized additional host responses including apoptosis, autophagy, iron metabolism, and thrombosis as investigational therapeutic targets.

*Staphylococcus aureus* is a common cause of community- and hospital-acquired infections, frequently complicated by the development of metastatic foci, recurrence despite prolonged antimicrobial treatment, and poor outcomes including death [1, 2]. Mortality associated with invasive *S aureus* infection has not improved significantly for several decades, and recent clinical trials of antimicrobial treatment strategies have failed to identify approaches that improve outcomes compared to standards of care [3]. Progressive acquisition of antimicrobial resistance further challenges our reliance on conventional antimicrobials. *Staphylococcus aureus* is well adapted to persist intracellularly including within professional phagocytes [4], protected from first-line β-lactams and glycopeptides [5], contributing to metastasis [6].

Therapeutic modulation of host responses against bacterial pathogens (host-directed therapies) represents a potential adjunct or alternative to conventional antimicrobials. Several examples demonstrate that augmenting host immunity is tractable [7]. For example, interferon gamma (IFN-γ) administration reduces the frequency of serious infections in people with chronic granulomatous disease [8] and can restore monocyte functionality in people with sepsis [9]. Similarly, treatment of critically ill adults with granulocyte-macrophage colony-stimulating factor reverses defective ex vivo neutrophil phagocytosis [10]. Host-directed therapies could also be applied to selectively modulate dysregulated inflammatory responses, or as adjuvants for vaccination.

Therapeutic targets for host-directed therapies for bacterial diseases remain largely elusive [7]. Host responses to infection are multilayered, incorporate redundancy, and are heterogeneous between individuals. We lack a granular understanding of the critical responses and their hubs that deterministically contribute to a signature of successful defense against specific pathogens. This contrasts with progress made in other fields, with the identification of targetable mutations in cancers, and “signature cytokines” in immune-mediated inflammatory diseases, informing precision immuno-therapeutic treatment [11]. Diverse and extensive sources of information are now available from genome-scale studies of host responses to bacterial infections. Integration of this information could provide a reformed basis for viewing host–pathogen interactions and prioritizing responses for investigation as data-driven therapeutic targets. To address this, we systematically aggregated and prioritized existing genome-scale data on the host response to *S aureus* in silico.

## METHODS

### Input Data

Inclusion and exclusion criteria were designed to identify genome-scale studies of host responses to *S aureus* and exclude candidate gene approaches to reduce bias (Supplementary Table 1). Accepted experimental methodologies were grouped into 5 categories: transcriptomics, proteomics, genome-wide association studies (GWASs), RNA interference screens, genome-wide CRISPR-Cas9 knockout screens, and epigenetics. The PubMed/Medline database was searched as shown in Supplementary Table 2. Manuscripts and supplemental files were reviewed to identify lists of host factors. Input lists were considered ranked if metrics of statistical significance and/or fold change were reported. These lists were ordered by *P* value/false discovery rate (low to high) then, where applicable, by absolute fold change or effect size (high to low). Gene names were converted to HUGO Gene Nomenclature Committee (HGNC) gene symbols (or Ensembl/Refseq symbols if no HGNC symbol). The National Center for Biotechnology Information Homologene database was used to map nonprimate genes to human homologues.

### Meta-analysis by Information Content

The meta-analysis by information content (MAIC) algorithm takes ranked or unranked lists of genes as input, classified by experimental category. Source code and documentation are available at https://github.com/baillielab/maic and the algorithm has been described and validated previously [12–15]. The output is a single aggregated ranked gene list. The ranking is based on a score determined by (1) the number of lists a gene appears in; (2) whether the lists a gene appears in contain a high proportion of overlap with other lists (as an indicator of list quality, exerting quality control); and (3) whether a gene is found in lists from different experimental methodology categories (reducing bias toward genes from lists from experimental categories more prevalent in the input).

MAIC considers each methodologic category equally so the methodology itself does not contribute to the ranking. The unit invariant knee method was used to identify prioritized genes with a MAIC score above the “elbow point” in the distribution of all scores, using the ARDSMAICR package in RStudio [16, 17].

### Functional Analyses

Cell- and tissue-specific enrichment of prioritized genes was performed using WebCSEA for the top 20 tissues and general cell types [18]. Preranked gene set enrichment analysis of prioritized genes was performed using the Fast Gene Set Enrichment Analysis package [19], with input ordered by MAIC score, using the Human MSigDB 50 Hallmark Gene Set, WikiPathways Human (2023), Gene Ontology Biological Processes (GO:BP, 2023), and Reactome (2023) databases. The Enrichr web interface [20–22] was used to identify traits from the UK Biobank GWAS catalogue [23] associated with prioritized genes. Immune cell type–specific expression of selected genes was determined using the Blood Atlas RNA sequencing dataset from the Human Protein Atlas [24], containing expression profiles for 18 peripheral blood immune cell populations and peripheral blood mononuclear cells. Differential gene expression was visualized using the *pheatmap* package (version 1.0.12) in RStudio. Protein–protein interactions (PPIs) were determined using STRING version 12 [25] in Cytoscape [26], including physical and functional interactions, and requiring a minimum interaction score of 0.7. Hub genes within the PPI network were identified by the maximum neighborhood component (MNC), maximal clique centrality, density of MNC, edge percolated component, and node degree methods, using cytoHubba, as previously described [27, 28].

### *S aureus* Bacteremia Cohort Study

Platelet counts and hemoglobin concentrations at the time of diagnosis of *S aureus* bacteremia (SAB) were available from an ongoing retrospective cohort study of adults with monomicrobial SAB conducted in Lothian, Edinburgh, United Kingdom (20 December 2019 to 4 November 2023, n = 690 patients). Details of an earlier analysis of this cohort have been previously published [2]. The South East Scotland Research Ethics Committee 02 approved this cohort study (23/SS/0025).

### A Priori Targets for Host-Directed Therapies

An a priori list of host factors that could be targeted using repurposed drugs was compiled (Supplementary Table 3) based on published literature reviews of host-directed therapies for any bacterial/mycobacterial pathogens, and review of more recent clinical and preclinical studies of host-directed therapies specifically for *S aureus* (see Supplementary References).

### Statistical Analysis

Platelet counts and hemoglobin concentrations were compared using a Kruskal-Wallis test with Dunn multiple comparisons test.

## RESULTS

### Meta-analysis by Information Content

**Figure 1.**
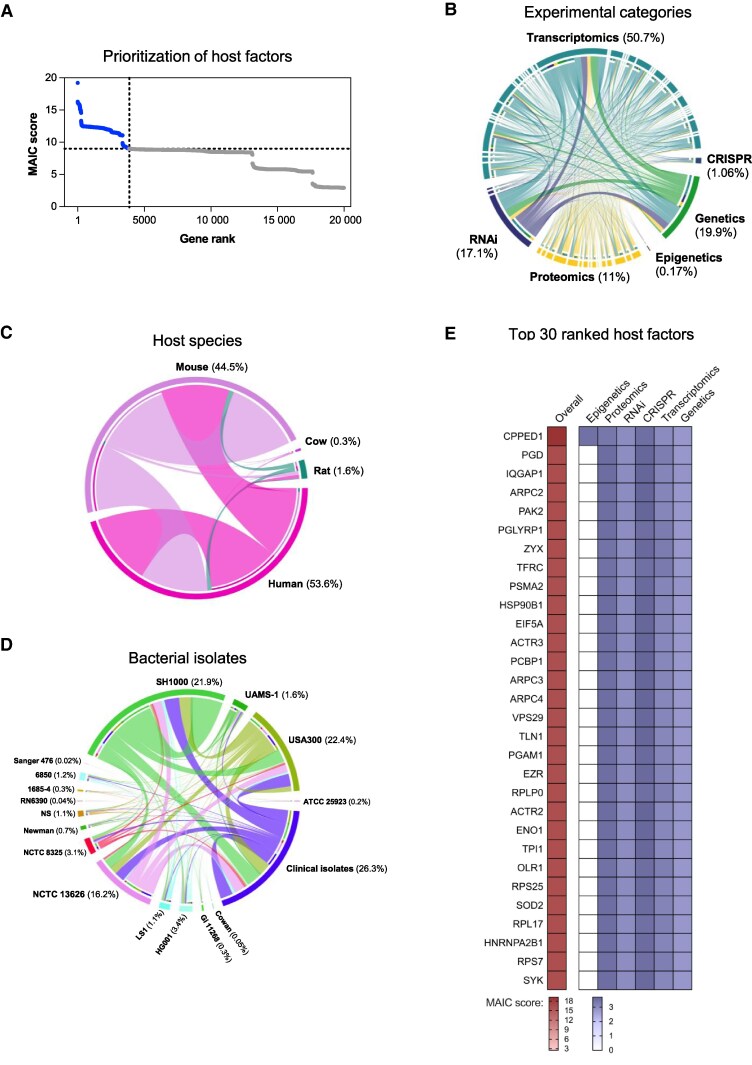
In silico prioritization of host factors. *A*, Distribution of meta-analysis by information content (MAIC) scores and ranks. Host factors prioritized by the analysis are shaded blue, determined using the unit invariant knee method. *B*, Shared information content between gene lists and weighting of experimental categories. Blocks on the outer ring represent gene lists, colored by experimental category, proportional in size to the contribution of that list to the total information content of the overall analysis. The total information content is the sum of gene scores from all included gene lists. The percentage refers to the percentage of the total information content that category contributed. The links in the plot indicate the sum of common gene scores between lists. Shared information content and weighting categorized by host species (*C*) and *Staphylococcus aureus* isolate (*D*) studied, where the outer blocks and colors represent host species or bacterial isolates, respectively. *E*, Heatmap of the highest-ranked 30 host factors showing the experimental categories contributing to the MAIC scores. Abbreviations: CRISPR, genome-wide CRISPR-Cas9 knockout screens; MAIC, meta-analysis by information content; RNAi, RNA interference screens.

Forty-seven eligible studies published between 2005 and 2023 were identified, from which 73 gene lists were retrieved and used as input for MAIC (Supplementary Figure 1, Supplementary Table 4). A total of 19 990 unique genes were identified, of which 3867 (19.3%) were prioritized by MAIC (Figure 1*A*). Prioritized genes were more likely to be present in multiple gene lists, and in lists from different experimental categories, compared to nonprioritized genes (Supplementary Figure 2). Transcriptomics studies contributed 50.7% of the final information content of the analysis (the total information content is the sum of gene scores from all included gene lists). Genetic studies contributed 19.9%, RNA interference 17.1%, proteomics 11.0%, CRISPR 1.1%, and epigenetics 0.2% (Figure 1*B*). There was a similar contribution of lists from studies of humans (53.6% information content) and nonhumans (46.4%; Figure 1*C*). Overlap between experimental methodologies, host species, and bacterial isolates was well distributed between input lists across the categories. The *S aureus* isolates investigated were varied, with the majority of the information content derived from studies using clinical isolates (26.3%), SH1000 (21.9%), and USA300 (22.4%) (Figure 1*D*). Monocytes, macrophages, and epithelial cells were the most enriched cell types among prioritized genes and the most enriched tissues included blood, spleen, and bone marrow (Supplementary Figure 3).

### Prioritized Host Responses

The top 30 prioritized genes are shown in Figure 1*F*. These include genes with functionally validated roles in defense against *S aureus*: superoxide dismutase 2 (*SOD2*, rank = 26) and spleen tyrosine kinase (*SYK,* rank = 30). *SOD2* is required for hydrogen peroxide–mediated intra-phagosomal killing of *S aureus* in macrophages [29]. *SYK* contributes to neutrophil phagocytosis and killing of *S aureus* [30]. Transferrin receptor (*TFRC*) was highly ranked (rank = 8) and controls intracellular iron levels and commitment to ferroptosis, an emerging cell death pathway with a potential role in macrophage defense against *S aureus* [31, 32]. Multiple other genes with potential roles in restricting iron availability to *S aureus* were also prioritized by MAIC (Supplementary Table 5) in addition to other genes regulating ferroptosis (eg, *PCBP1*, rank = 13; *HSPA8*, rank = 46; *HSPA5*, rank = 70; *ATG3*, rank = 861; *HAMP*, rank = 1972) [33]. In a cohort of adults with SAB, we found baseline hemoglobin concentration (as a proxy marker of iron availability) was higher in those with endocarditis or other metastatic complications, compared to uncomplicated bacteremia Supplementary Figure 4).

**Figure 2.**
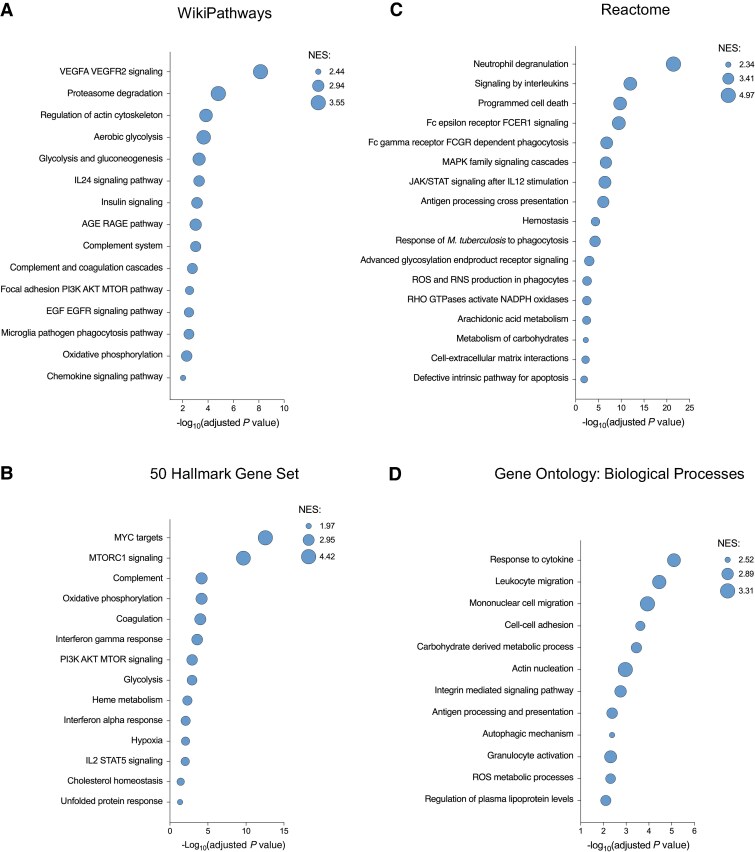
Gene set enrichment analysis using prioritized genes. Enriched terms/classes among prioritized host genes, using the WikiPathways Human (2023) (*A*), 50 Hallmark Gene Set (*B*), Reactome (2023) (*C*), and Gene Ontology Biological Processes (2023) databases (*D*). Bubble size proportional to normalized enrichment score (NES).

The full list of prioritized genes is available in the Supplementary Material. Many genes underlying inherited or acquired susceptibility to *S aureus* infection were prioritized by the analysis (Table 1). Gene set enrichment analysis of prioritized genes identified pathways related to myeloid cell antimicrobial responses (including IFN-γ specifically), glucose metabolism, apoptosis, autophagy, the proteasome, and coagulation (Figure 2). Proteasome activator complex subunit 3 (*PSME3*) was highly ranked (rank = 266) and recently demonstrated to coordinate the production of novel proteasome-derived antibacterial defense peptides [34], including *PPP1CB*, *DCTN4*, *PSMG2*, and *RPS4X*, which were also prioritized by MAIC.

**Table 1.** Genes Involved in Monogenic Susceptibility to *Staphylococcus aureus* Infection Prioritized by Meta-analysis by Information Content

Syndrome	Gene	MAIC Rank^a^	Reference
Autosomal dominant hyper-IgE syndrome	*STAT3*	354	[46]
	*IL6ST*	1597	
Anti-IL-6 autoantibodies	*IL6*	3789	[47]
Chronic granulomatous disease	*CYBB (gp91^phox^)*	3375	[48]
	*CYBA (p22^phox^)*	348	
	*NCF1 (p47^phox^)*	3477	
	*NCF2 (p67^phox^)*	770	
Autosomal recessive IL-17RA deficiency	*IL17A*	3774	[49]
	*IL17RA*	547	
Heterozygous STAT1 gain-of-function	*STAT1*	983	[50]

Abbreviations: IgE, immunoglobulin E; IL, interleukin; MAIC, meta-analysis by information content.

^a^Ranking out of 3876 prioritized genes.

### Hubs in Effector Responses

**Figure 3.**
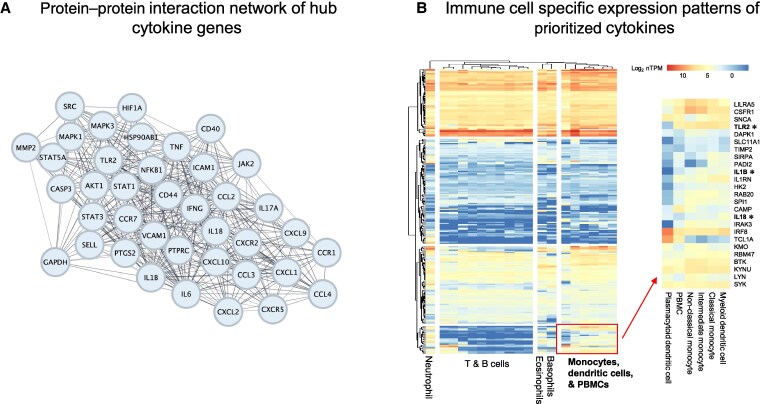
Hubs in the cytokine response. *A*, Protein–protein interaction network of hub cytokines prioritized by meta-analysis by information content from the Gene Ontology Biological Processes (GO:BP) “cytokine” term. *B*, Clustered heatmap of immune cell type messenger RNA expression patterns of prioritized genes in GO:BP “cytokine” term. Gene names in bold font marked with *indicate hub cytokines. Abbreviations: MAIC, meta-analysis by information content; PBMC, peripheral blood mononuclear cell.

We identified cytokine signaling, apoptosis, and autophagy as effector responses with relevance to clearance of persistent intracellular *S aureus*, a recognized challenge to successful antimicrobial treatment. To investigate the regulation of these 3 processes and identify potential points for intervention, we identified hub genes within networks of predicted PPIs of prioritized genes from the GO:BP “cytokine” and “autophagic mechanism” terms, and the Reactome “programmed cell death” term. Thirty-nine hub cytokines were identified (Figure 3*A*) including IFN-γ, interleukin (IL) 18, IL-1β, IL-6, and IL-17A. As monocyte and macrophage gene expression was enriched among the entire set of prioritized genes, we also defined monocyte/macrophage-specific cytokine expression (Figure 3*B*). This identified a cluster of genes specifically expressed by monocytes/peripheral blood mononuclear cells/dendritic cells, including colony-stimulating factor 1 receptor (*CSF1R*), multiple autophagy regulators (*DAPK1*, *RBM47*, *LYN*, and *SIRPA*), and the antimicrobial peptide LL-37 (*CAMP*). Three of these genes, *TLR2*, *IL-1β*, and *IL18*, were also hub cytokines identified in our PPI analysis. Thirty hub genes related to apoptosis were identified, including *BAD*, *CASP3*, *CASP8*, and *CYCS* (Supplementary Figure 4*A*). Twenty-seven hub genes related to autophagy were identified, including *MTOR*, *HIF1A*, and *LAMP2* (Supplementary Figure 4*B*).

### Targets for Repurposed Drugs

**Figure 4.**
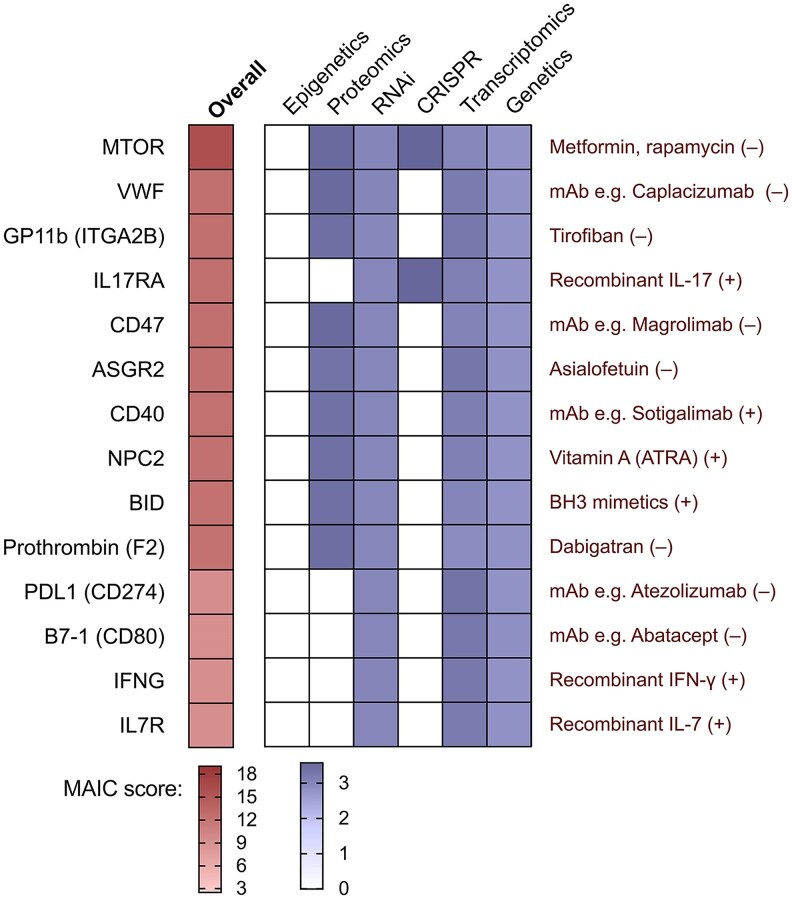
Therapeutic targets. Heatmap of targets for potential drug repurposing prioritized by meta-analysis by information content (MAIC), showing the experimental categories contributing to the MAIC scores for the host factor targets. Candidate drugs are shown in red font; “+” indicates the drug is an agonist and “–“ indicates the drug is an inhibitor. Abbreviations: ATRA, all-trans retinoic acid; CRISPR, genome-wide CRISPR-Cas9 knockout screens; mAb, monoclonal antibody; MAIC, meta-analysis by information content; RNAi, RNA interference screens.

MAIC scores were retrieved for an a priori list of potential therapeutic targets (Supplementary Table 3). Of 23 direct targets for potential drug repurposing, 14 were prioritized by our analysis including several hub genes (Figure 4). These included targets related to inhibition of *S aureus*/fibrin/platelet microthrombus formation (VWF, GP11b), inhibition of *S aureus*–induced platelet loss (ASGR2), and modulation of immune responses. These responses included host factors involved in antimicrobial effector functions including intracellular bacterial killing (IL-17RA, IL-7R, IFN-γ), apoptosis (BID), autophagy (mTOR), and immune checkpoints (PDL1).

### Bacteria–Platelet Interactions

**Figure 5.**
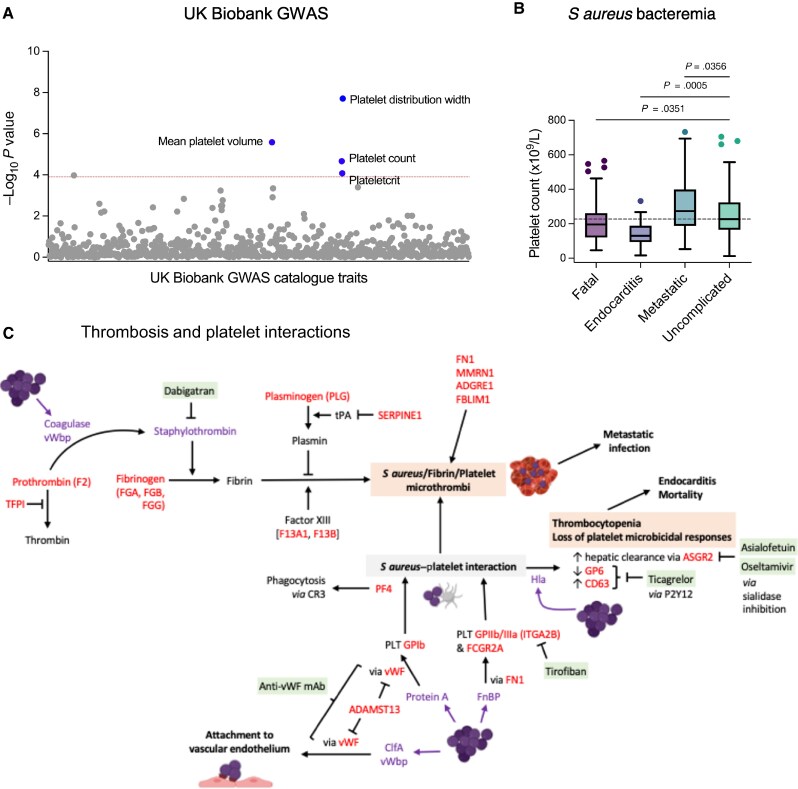
Platelets and thrombosis in the host response to *Staphylococcus aureus*. *A*, Manhattan plot of traits in the UK Biobank genome-wide association study (GWAS) catalogue associated with genes prioritized by meta-analysis by information content (MAIC). Horizontal red broken line indicates adjusted *P* value <.05. *B*, Platelet counts in adults with *S aureus* bacteremia stratified by outcome of infection. Outcome was assigned in the following hierarchy: fatal > endocarditis > other metastatic infection. Uncomplicated bacteremia refers to patients surviving to hospital discharge with no clinically apparent metastatic foci. Box and whisker plot drawn using Tukey method. Box shows interquartile range and horizontal line shows median. Horizontal broken line indicates median platelet count for uncomplicated bacteremia. Multiple comparisons made to uncomplicated bacteremia as control group; adjusted *P* values shown. *C*, Host factors and therapeutic targets involved in formation of *S aureus*/fibrin/platelet microthrombi and *S aureus*–induced thrombocytopenia. Purple text indicates *S aureus* factors. Host factors in red are prioritized by MAIC. Green shading indicates potential therapeutic interventions.

In an analysis of the UK Biobank GWAS catalogue, prioritized genes were associated with platelet count and platelet morphology (Figure 5*A*). To determine if this was clinically relevant, we examined baseline platelet count during SAB, finding that platelet count was associated with the outcome of bacteremia (Figure 5*B*). Compared to people with uncomplicated bacteremia, platelet counts were lower in people with fatal disease or endocarditis, and higher in people with metastatic infections (excluding endocarditis). Cross-referencing prioritized genes with known biology relating to *S aureus* interactions with thrombosis and platelets identified relevant host factors prioritized in our analysis [35]. This also supported 5 points of potential therapeutic intervention in this process that could inhibit bacteria/fibrin/platelet microthrombus formation or prevent accelerated platelet clearance (Figure 5*C*).

## DISCUSSION

The heterogeneity and interpretive complexity of pathogen–host interactions are significant barriers to the identification of key processes and hub factors that can be investigated as targets for host-directed therapies. Our in silico analysis provides a solution to this problem by systematically aggregating and prioritizing the extensive and diverse existing genome-scale data on the host response to *S aureus*. Our chosen method, MAIC, has already been validated and shown to perform better than other ranking aggregation methods for this purpose [12, 13].

Confidence in our analysis is enhanced by the unbiased recapitulation of established and validated biology, in particular the prioritization of genes involved in monogenic susceptibility to *S aureus* (Table 1). Our findings prioritize specific elements of innate immune antimicrobial effector responses, including IL-17, IL-1β, and IFN-γ, which are all supported by preexisting functional or clinical data. IL-17 promotes bacterial clearance in a mouse model of *S aureus* skin infection [36], IL-1β is associated with successful clearance of bacteremia in humans with SAB [37], and administration of IFN-γ protects against staphylococcal infections in people with chronic granulomatous disease [8]. This provides confidence in the utility of the MAIC output and supports the investigation of other prioritized processes that are noncanonical and have been less extensively investigated. Specifically, prioritized genes and pathways indicate that regulation of apoptosis, the proteasome, autophagy, and iron metabolism should be prioritized for investigation. *Staphylococcus aureus* infection is associated with increased macrophage expression of anti-apoptotic *MCL1*, and failure of apoptosis-associated bacterial killing, which normally provides a default clearance mechanism for other pathogens when phagolysosomal killing has been exhausted [38, 39]. Approaches to reengage this defense mechanism could augment clearance of intracellular *S aureus.* Therapeutic induction of macrophage apoptosis has been shown to enhance bacterial clearance in mouse models of pneumonia caused by *Streptococcus pneumoniae*, *Haemophilus influenzae*, and *Legionella longbeachae* using BH3 mimetics; however, this specific approach failed to improve clearance of *S aureus* [38, 40]. The proteasome has a recognized role in antigen presentation during infection but a role in direct bacterial killing has only very recently been described, coordinated by PSME3 and effected by proteasome cleaved antimicrobial peptides including PPP1CB, both prioritized in our analysis [34]. This microbicidal role has been investigated using *Micrococcus luteus* and *Staphylococcus haemolyticus* but not yet *S aureus*. Autophagy represents a potential therapeutic target through modulation of mTOR, and has a well-described role in clearance of the classical intracellular pathogens *Listeria monocytogenes* and *Mycobacterium tuberculosis* [41]. Augmenting natural nutritional immunity by restricting bacterial iron acquisition has previously been demonstrated to improve bacterial clearance in mice after intravenous *S aureus* inoculation [42]. The prioritization of several genes involved in iron sequestration, and the unexpectedly higher hemoglobin concentrations in people with metastatic SAB, further supports investigating this, in addition to the emerging cell death pathway of ferroptosis [33].

There is current interest in the modulation of thrombosis and platelet responses as an adjunct to antimicrobials in SAB, including a case report of ticagrelor adjunctive therapy in a person with persistent SAB associated with endovascular infection and metastatic foci [43]. Our analysis supports therapeutic targeting of mechanisms associated with thrombocytopenia (for example ticagrelor) and bacteria/fibrin/platelet microthrombus formation, for example, using dabigatran. Differential treatment effects are likely to exist between endotypes of *S aureus* disease, as has been demonstrated for adjunctive rifampicin [44]. Low platelet counts were associated with endocarditis, suggesting preventing platelet loss could be beneficial. In contrast, higher counts were associated with musculoskeletal metastatic infection (without endocarditis), suggesting that inhibiting microthrombus formation could be more beneficial.

There are important limitations to this study. First, the ranking is based on the strength of evidence for any association with *S aureus* disease and cannot distinguish between pro- or antibacterial effects of a given host factor, or roles in dysregulated immunity. For example, C5AR1 was a prioritized gene and activation by C5a impairs neutrophil phagolysosomal killing of *S aureus*, contributing to immune failure during critical illness [45]. Second, included studies were heterogeneous in terms of the model systems and bacterial strains used. While this is also a strength that provides a global view of the host response, it means the final ranking applies at the broadest level. Consideration of cell or tissue types of interest, and use of several bacterial strains, will be required when planning functional validation. Finally, lack of evidence from genome-scale studies does not exclude important roles. For example, IL-10 is reproducibly associated with mortality in SAB but was not prioritized by MAIC [37].

In conclusion, we have systematically aggregated results from genome-scale studies of the host response to *S aureus*, generating an in silico integrated and prioritized list of host factors with the strongest cumulative evidence. This identifies hubs in the immune response and prioritizes the investigation of bacteria–platelet interactions, apoptosis, autophagy, iron metabolism and ferroptosis, and the proteasome as targets for host-directed therapies.

## Supplementary Material

jiaf290_Supplementary_Data

## Supplementary Data

Supplementary materials are available at *The Journal of Infectious Diseases* online (http://jid.oxfordjournals.org/). Supplementary materials consist of data provided by the author that are published to benefit the reader. The posted materials are not copyedited. The contents of all supplementary data are the sole responsibility of the authors. Questions or messages regarding errors should be addressed to the author.

## Notes

***Author contributions*.** C. D. R., J. K. B., and D. H. D. conceived the study. C. D. R., S. G.-T., and E. S. curated the data. C. D. R., J. E. M., N. P., S. H. C., M. S., and B. W. did the formal analysis. C. D. R. supervised the study, validated the study data, and wrote the original draft of the manuscript. All authors reviewed and edited the manuscript. C. D. R., S. G.-T., E. S., and B. W. had access to the raw data. The corresponding author had full access to all the data and final responsibility for the decision to submit for publication.

***Data availability*.** The MAIC output is included as Supplementary Material.

***Financial support.*** C. D. R. was supported by an Edinburgh Clinical Academic Track/Wellcome Trust PhD Training Fellowship for Clinicians award (214178/Z/18/Z) and by the Academy of Medical Sciences (SGL032\1020). N. P. was supported by the Wellcome Trust (211496/Z/18/Z). D. H. D. was supported by UK Research and Innovation/Medical Research Council (MRC) (program grant MR/Y013131/1). This work was supported by antimicrobial resistance cross-council funding from the MRC to the SHIELD consortium “Optimising Innate Host Defence to Combat Antimicrobial Resistance” (MR/NO2995X/1; principal investigator: D. H. D.).
