# Supplementary material for: Genome-Scale Meta-analysis of Host Responses to Staphylococcus aureus Identifies Pathways for Host-Directed Therapeutic Targeting
Source: J Infect Dis. 2025 May 31;232(2):e290–300. doi: 10.1093/infdis/jiaf290 (PMC12349957; doi:10.1093/infdis/jiaf290)
Supplement: jiaf290_Supplementary_Data [file jiaf290_supplementary_data.pdf]

Supplementary Material

Genome-scale meta-analysis of host responses to *Staphylococcus aureus* identifies pathways for host-directed therapeutic targeting

Figure S1: PRISMA flow diagram of systematic review. 2

Figure S2: Comparison of prioritised and non-prioritised genes. 3

Figure S3: Cell and tissue enrichment of prioritised genes. 4

Figure S4: Baseline haemoglobin concentration in adults with *S. aureus* bacteraemia. 5

Figure S5: Apoptosis and autophagy hub genes. 6

Table S1: Inclusion and exclusion criteria 7

Table S2: Search terms 8

Table S3: *A priori* list of targets for host-directed therapies 9

Table S4: Sources of input data 10

Table S5: Prioritised genes involved in iron metabolism 14

References 15

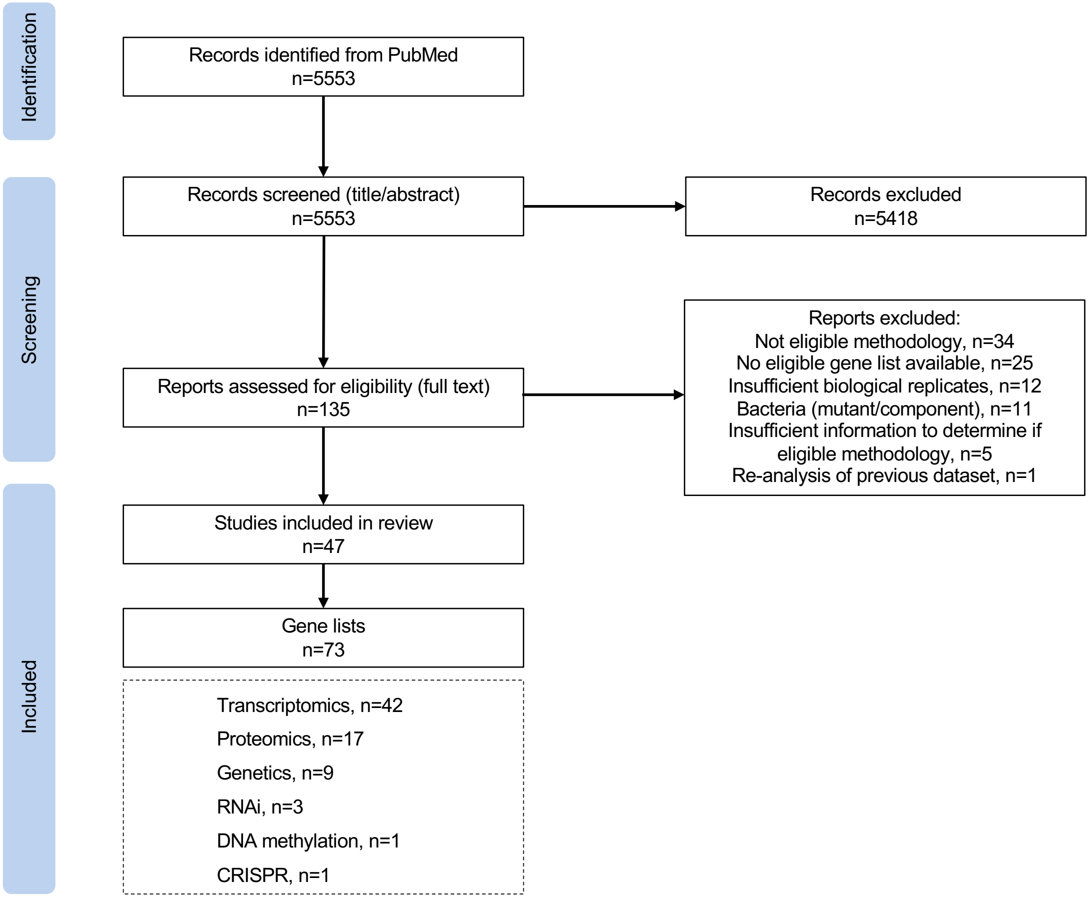

Figure S1: PRISMA flow diagram of systematic review.

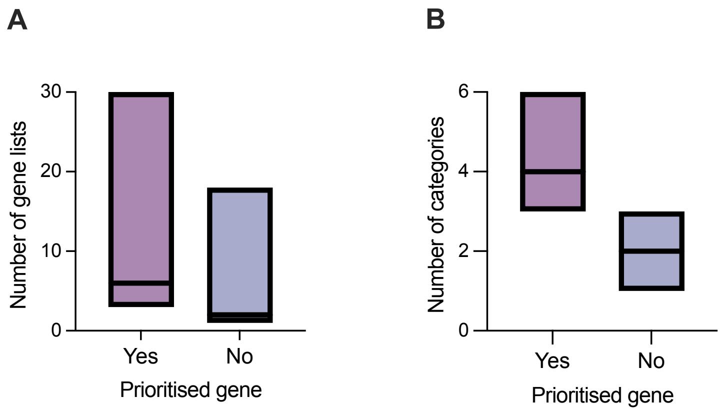

Figure S2: Comparison of prioritised and non-prioritised genes.
(A) Number of gene lists including the gene. **(B)** Number of experimental categories gene identified by. Horizontal line shows median, box shows minimum to maximum.

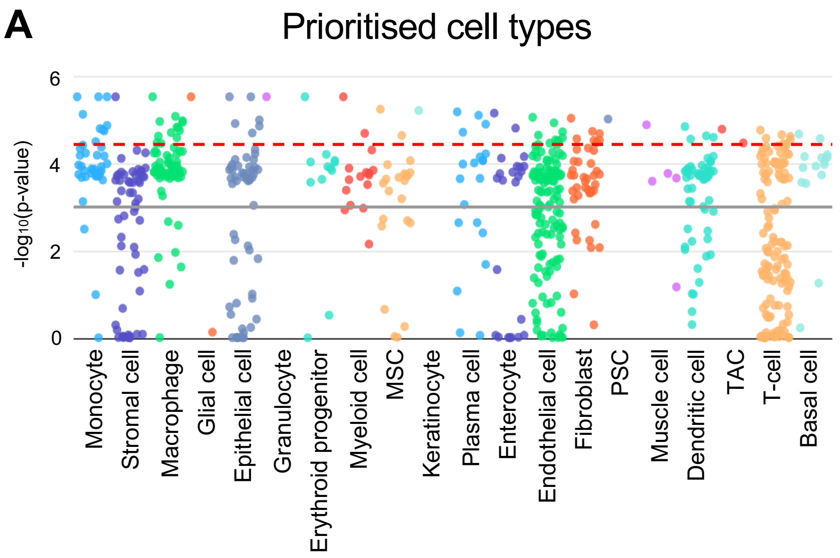

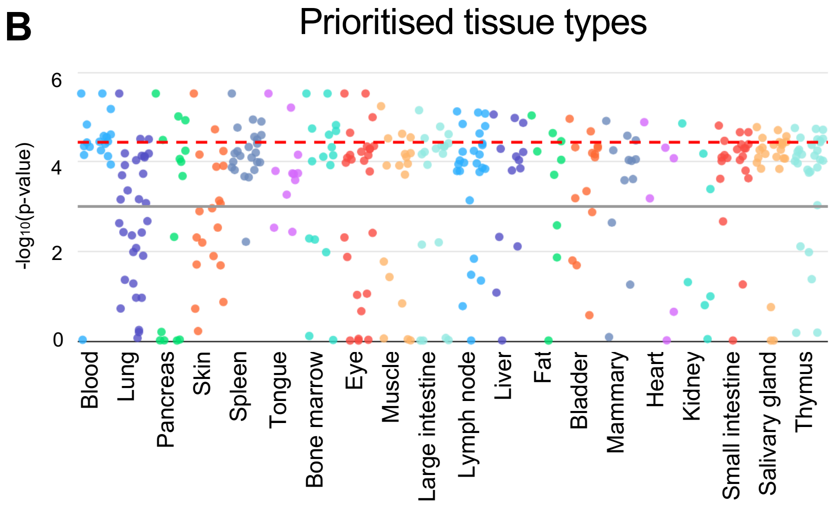

Figure S3: Cell and tissue enrichment of prioritised genes.
Manhattan plot of the top 20 **(A)** cell and **(B)** tissue types enriched for expression of prioritised genes. Horizontal solid grey line indicates nominal statistical significance (p=0.001). Horizontal broken red line indicates Bonferroni-corrected p=0.001 (raw p=3.69×10^{-5}).

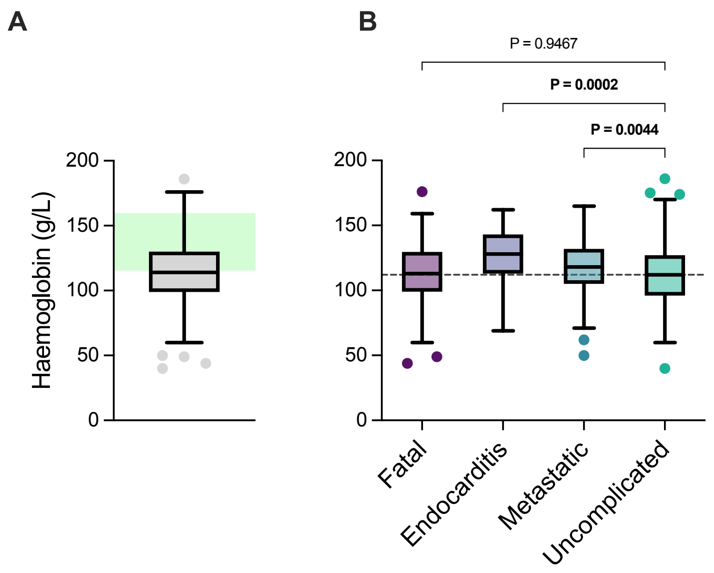

Figure S4: Baseline haemoglobin concentration in adults with *S. aureus* bacteraemia.
(A) Distribution of baseline haemoglobin measurements in all included patients (n=690). Green shaded area represents reference range. **(B)** Haemoglobin measurements stratified by outcome of infection. Outcome was assigned in the following hierarchy: fatal (attributable in-hospital mortality) > endocarditis > other metastatic infection. Uncomplicated bacteraemia refers to patients surviving to hospital discharge with no clinically-apparent metastatic foci. Box and whisker plot drawn using Tukey's method. Box shows interquartile range and horizontal line shows median. Horizontal broken line indicates median haemoglobin concentration for uncomplicated bacteraemia. Multiple comparisons made to uncomplicated bacteraemia as control group; adjusted p-values shown.

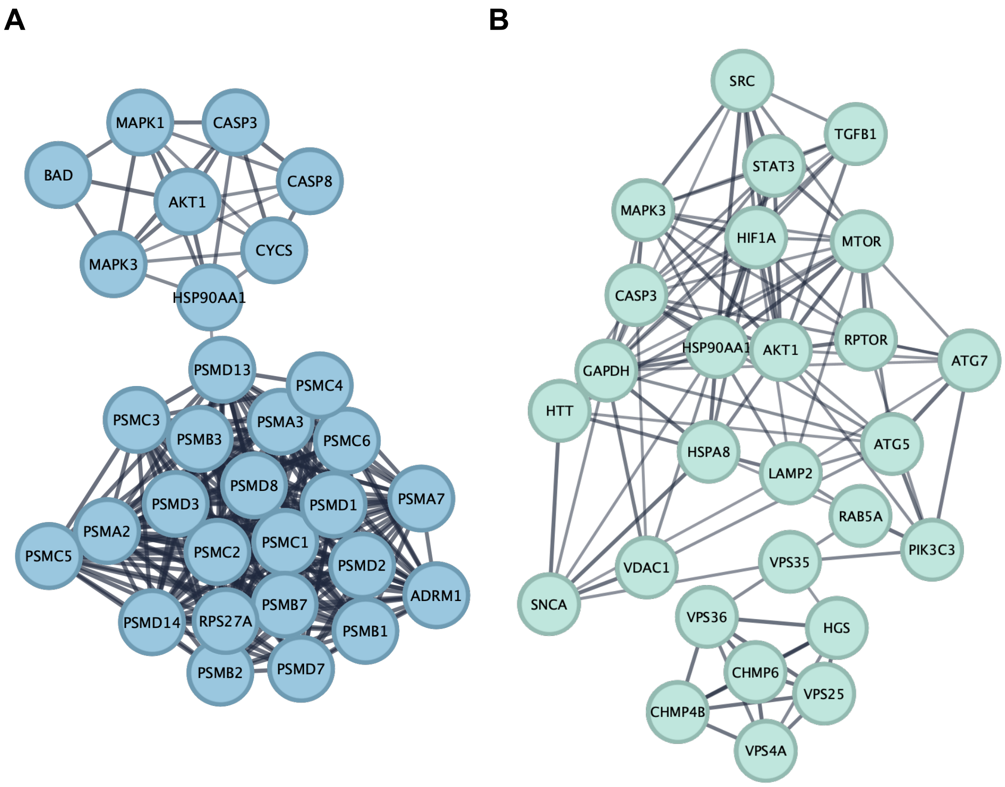

Figure S5: Apoptosis and autophagy hub genes.
Protein-protein interaction networks of hub genes prioritised by MAIC from **(A)** the Reactome "Programmed cell death" term and **(B)** the GO:BP "Autophagic mechanism" term.

Table S1: Inclusion and exclusion criteria

Inclusion	Exclusion
• Host factors involved in *S. aureus* infection<br>• Accepted genome-scale experimental methodologies<br>• Live bacteria<br>• *In vitro*/*ex vivo* studies of primary human or wild type animal cells/tissues<br>• *In vitro* study of un-edited cell lines<br>• *In vivo* studies of infected humans or wild type animal infection models	• Studies reporting only bacterial factors<br>• Clinical studies with <5 biological replicates<br>• Candidate studies (<50 genes/proteins)<br>• Studies using mutant bacteria, or only using bacterial components (e.g. toxins)<br>• Studies of animal diseases not a model of human disease (*S. aureus* bovine mastitis)<br>• Re-analysis of previous dataset<br>• Incomplete data available for gene list preparation*

* If only a non-systematically selected sub-set of genes was available (e.g. a volcano plot annotated with selected genes) the study was excluded.

Table S2: Search terms

(“staphylococcus aureus” OR “MRSA” OR “MSSA” OR “S* aureus”) AND ((transcript* [Title/Abstract]) OR (proteom*[Title/Abstract]) OR (siRNA[Title/Abstract]) OR (shRNA [Title/Abstract]) OR (CRISPR*[Title/Abstract]) OR (“rnaseq”[Title/Abstract]) OR (rna-seq[Title/Abstract]) OR (“scRNA-seq”[Title/Abstract]) OR (microarray[Title/Abstract]) OR (“genome wide association study”[Title/Abstract]) OR (“affymetrix” [Title/Abstract]))

Search performed on 14th February 2023 without any date or language restrictions

Table S3: ***A priori*** **list of targets for host-directed therapies**

Process	Host factor	Drug	Ref.
**Inhibition of *S. aureus*/fibrin/platelet microthrombus formation**	VWF	mAb Caplacizumab	[1,2]
	F2 (prothrombin)	Dabigatran (inhibitor)	[3-5]
	GP11b (ITGA2B)	Tirofiban, abciximab (inhibitors)	[5,6]
**Inhibition of *S. aureus*-induced platelet loss**	P2Y12	Ticagrelor, ticlodipine (inhibitors)	[5,7,8]
	ASGR2	Asialofetuin (hepatic Ashwell-Morell receptor inhibitor)	[8]
	NEU1	Sialidase inhibitors:<br>Oseltamivir<br>Neu1-selective sialidase inhibitor C9-butyl-amide-2-deoxy-2,3-dehydro-N-acetylneuraminic acid	[8]
**Augmentation of host anti-microbial immune responses**	MTOR	Metformin, rapamycin	[9]
	IL17RA	Recombinant IL-17	[10,11]
	CD47	Anti-CD47 mAb	[12]
	CD40	Agonist mAb (e.g. Sotigalimab)	[9]
	NPC2	Vitamin A (ATRA)	[9]
	BID	BH3 mimetics	[10]
	PDL1 (CD274)	Anti-PD-L1 mAb (e.g. Atezolizumab)	[9,10]
	PD1	Anti-PD-1 mAb (e.g. Nivolumab)	[9,10]
	B7-1 (CD80)	Abatacept (inhibitor)	[9]
	IFNG	Recombinant IFNG	[10]
	IL7R	Recombinant IL-7	[9,10]
	GM-CSF	Recombinant GM-CSF	[10]
	NFE2L2	Nrf2 agonists	[10]
	CTLA4	Anti CTLA-4 mAb	[9,10]
	PI3K	PI3K inhibitors	[9,10,13]
	LAG3	Anti-LAG3 mAb	[9]
	TIM3	Anti-TIM3 mAb	[9]

Table S4: Sources of input data

Study	Year	Methodology	Species	Infection	Tissue	Cell type	*S. aureus* isolate	Focus
Matussek[14]	2005	Transcriptomics	Human	*In vitro*	–	Endothelial (HUVEC)	Clinical	Infected vs. uninfected
Moreilhon[15]	2005	Transcriptomics	Human	*In vitro*	–	Epithelial (MM-39)	SH1000	Infected vs. uninfected
Ardura[16]	2009	Transcriptomics	Human	*In vivo*	Blood	PBMC	Clinical	Infected vs. uninfected
Stark[17]	2009	Transcriptomics	Human	*In vitro*	–	Endothelial (HUVEC)	Clinical	Infection with bacteraemia vs. nasal carriage isolates
Li[18]	2009	Transcriptomics	Human	*In vitro*	–	Epithelial (Hep-2)	RN6390	Infected vs. uninfected
Kobayashi[19]	2010	Transcriptomics	Human	*In vivo*	Blood	Neutrophil	Clinical	Infection with community-associated vs. healthcare associated MRSA isolates
Grundmeier[20]	2010	Transcriptomics	Human	*In vitro*	–	Endothelial (HUVEC)	Cowan	Infection with Cowan vs. other strains
Tchatalbachev[21]	2010	Transcriptomics	Human	*In vitro*	Blood	Monocyte (CD14+)	Gi.11268	Infected vs. uninfected
Ahn[22]	2010	Genetics	Mouse	*In vivo*	–	–	Sanger 476	Infection of differentially susceptible mice (A/J vs. C57BL/6J)
Banchereau[23]	2012	Transcriptomics	Human	*In vivo*	Blood	–	Clinical	Disease phenotype: pneumonia vs. osteoarticular infection
Sabat[24]	2013	Transcriptomics	Cow	*In vitro*	Blood	MDM	1685-4	Infected vs. uninfected
Nelson[25]	2014	Genetics	Human	*In vivo*	–	–	Clinical	Susceptibility: acquisition of SAB in healthcare setting
Ye[26]	2014	Genetics	Human	*In vivo*	–	–	Clinical	Susceptibility: *S. aureus* disease
Agudelo[27]	2014	Transcriptomics	Human	*In vivo*	Blood	–	Clinical	Infected (MRSA haematogenous osteomyelitis) vs. uninfected
Yan[28]	2014	Genetics	Mouse	*In vivo*	–	–	Sanger 476	Infection of differentially susceptible mice (A/J vs. C57BL/6J)
Brown[29]	2015	Genetics	Human	*In vivo*	–	–	Clinical	Susceptibility: *S. aureus* nasal carriage
Brady[30]	2015	Transcriptomics	Mouse	*In vivo*	Ear	–	USA300	Infected vs. uninfected (day 1)
		Transcriptomics	Mouse	*In vivo*	Ear	–	USA300	Infected vs. uninfected (day 7)
Delorenze[31]	2016	Genetics	Human	*In vivo*	–	–	Clinical	Susceptibility: *S. aureus* disease
Yan[32]	2017	Genetics	Mouse	*In vivo*	–	–	Sanger 476	Infection of differentially susceptible mice (A/J vs. C57BL/6J)
Thanert[33]	2017	Transcriptomics	Mouse	*In vivo*	Kidney	–	SH1000	Infected vs. uninfected (A/J mice)

		Transcriptomics	Mouse	*In vivo*	Kidney	–	SH1000	Infected vs. uninfected (C57BL/6J mice)
Lei[34]	2017	Proteomics	Rat	*In vivo*	Orthopaedic implant	–	UAMS-1	Infected vs. uninfected (day 6, secretome)
		Proteomics	Rat	*In vivo*	Orthopaedic implant	–	UAMS-1	Infected vs. uninfected (day 6, surfactome)
		Proteomics	Rat	*In vivo*	Orthopaedic implant	–	UAMS-1	Infected vs. uninfected (day 45, secretome)
		Proteomics	Rat	*In vivo*	Orthopaedic implant	–	UAMS-1	Infected vs. uninfected (day 45, surfactome)
Moreau[35]	2018	Genetics	Human	*In vivo*	–	–	Clinical	Disease phenotype: SAB with vs. without native valve endocarditis
Scott[36]	2018	Genetics	Human	*In vivo*	–	–	Clinical	Disease phenotype: complicated vs. uncomplicated SAB
Chen[37]	2018	Transcriptomics	Human	*In vivo*	Blood	–	Clinical	Infected (any *S. aureus* infection) vs. uninfected
		Transcriptomics	Human	*In vivo*	Blood	–	Clinical	Infected (*S. aureus* osteomyelitis) vs. uninfected
		Transcriptomics	Human	*In vivo*	Blood	–	Clinical	Infected (*S. aureus*, not osteomyelitis) vs. uninfected
Sun[38]	2018	Transcriptomics	Human	*In vitro*	Blood	MDM	Newman	Infected vs. uninfected (8h)
		Transcriptomics	Human	*In vitro*	Blood	MDM	Newman	Infected vs. uninfected (24h)
		Transcriptomics	Human	*In vitro*	Blood	MDM	Newman	Infected vs. uninfected (48h)
Medina[39]	2019	Proteomics	Human	*In vitro*	–	Epithelial (16HBE14o-)	HG001	Later infection timepoints vs. 0h timepoint
Bravasantano[40]	2019	RNAi screen	Human	*In vitro*	–	Epithelial (HeLa)	NCTC 13626	*S. aureus*-induced host cell death
Toledo[41]	2019	Proteomics	Mouse	*In vivo*	Brain	–	USA300	Infected vs. uninfected
		Proteomics	Mouse	*In vivo*	Heart	–	USA300	Infected vs. uninfected
		Proteomics	Mouse	*In vivo*	Kidney	–	USA300	Infected vs. uninfected
		Proteomics	Mouse	*In vivo*	Liver	–	USA300	Infected vs. uninfected
Jacquet[42]	2019	Transcriptomics	Mouse	*In vivo*	Skin	–	USA300	Infection of control vs. streptozotocin-induced hyperglycaemic mice
Stelzner[43]	2020	RNAi screen	Human	*In vitro*	–	Epithelial (HeLa)	6850	*S. aureus*-induced cytotoxicity
		RNAi screen	Human	*In vitro*	–	Epithelial (HeLa)	6850	Intracellular invasion
Michalik[44]	2020	Proteomics	Human	*In vivo*	Blood	–	Clinical	Infected (SAB) vs. uninfected

Wozniak[45]	2020	Proteomics	Human	*In vivo*	Blood	–	Clinical	Infected (SAB) vs. uninfected
		Proteomics	Human	*In vivo*	Blood	–	Clinical	Outcome of SAB: survival vs. death
Chang[46]	2021	Epigenetics	Human	*In vivo*	Blood	Leukocyte	Clinical	Disease phenotype: persistent vs. resolving SAB
Xiao[47]	2021	Transcriptomics	Human	*In vivo*	Blood	–	Clinical	Infected (*S. aureus* infection) vs. uninfected
		Transcriptomics	Human	*In vivo*	Blood	–	Clinical	Disease phenotype: *S. aureus* disease with vs. without endocarditis
Lin[48]	2021	Transcriptomics	Mouse	*In vivo*	Bone	–	Clinical	Infected vs. uninfected (day 3)
		Transcriptomics	Mouse	*In vivo*	Bone	–	Clinical	Infected vs. uninfected (day 14)
Lindner[49]	2021	CRISPR screen	Human	*In vitro*	–	Monocyte (THP-1)	NS	Phagocytosis of *S. aureus*
Zhang[50]	2022	Transcriptomics	Mouse	*In vivo*	Skin	–	ATCC 25923	Infected vs. uninfected
Chen[51]	2022	Proteomics	Human	*In vivo*	Articular fluid	–	Clinical	Infected vs. uninfected
Garcia-Moreno[52]	2022	Proteomics	Mouse	*In vitro*	–	Osteoblast (MC3T3-E1)	LS1	Infected vs. uninfected
		Proteomics	Mouse	*In vitro*	–	Osteocyte (MLO-Y4)	LS1	Infected vs. uninfected
Nicolas[53]	2022	Transcriptomics	Human	*In vitro*	–	Osteoblast (MG-63)	NCTC 8325	Infected vs. uninfected
Goldmann[54]	2022	Transcriptomics	Human	*In vitro*	–	Mast cell (HMC-1)	SH1000	Infected vs. uninfected
Dietrich[55]	2022	Transcriptomics	Mouse	*In vivo*	Spleen	Monocyte (classical)	SH1000	Infected vs. uninfected
		Transcriptomics	Mouse	*In vivo*	Spleen	Monocyte (non-classical)	SH1000	Infected vs. uninfected
		Transcriptomics	Mouse	*In vivo*	Spleen	Dendritic cell	SH1000	Infected vs. uninfected
		Transcriptomics	Mouse	*In vivo*	Spleen	B-cell	SH1000	Infected vs. uninfected
		Transcriptomics	Mouse	*In vivo*	Spleen	T-cell	SH1000	Infected vs. uninfected
		Transcriptomics	Mouse	*In vivo*	Spleen	Natural killer cell	SH1000	Infected vs. uninfected
		Transcriptomics	Mouse	*In vivo*	Spleen	Plasma cell	SH1000	Infected vs. uninfected
		Transcriptomics	Mouse	*In vivo*	Spleen	Neutrophil	SH1000	Infected vs. uninfected
Muller[56]	2023	Transcriptomics	Human	*In vitro*	Blood	Monocyte	6850	Infected vs. uninfected
		Transcriptomics	Human	*In vitro*	Blood	Monocytes	Clinical	Infected (SAB) vs. uninfected
Sirichoat[57]	2023	Proteomics	Human	*In vitro*	Blood	Leukocyte	ATCC 25923	Infected vs. uninfected
Fisher[58]	2023	Proteomics	Human	*In vivo*	Articular fluid	–	Clinical	Infected (PJI) vs. uninfected (non-infectious arthroplasty failure)

Thaden[59]	2023	Transcriptomics	Human	*In vivo*	Blood	–	Clinical	Infected (SAB) vs. uninfected (gram-negative bacteraemia)
		Transcriptomics	Human	*In vivo*	Blood	–	Clinical	MSSA SAB vs. MRSA SAB
Zhao[60]	2023	Transcriptomics	Mouse	*In vivo*	Lung	–	USA300	Infected vs. uninfected

HUVEC: human umbilical vein endothelial cell; PBMC: peripheral blood mononuclear cell; MDM: monocyte-derived macrophage; SAB: Staphylococcus aureus bacteraemia.

Table S5: Prioritised genes involved in iron metabolism

Gene	MAIC score	MAIC rank	Function
Transferrin receptor (*TFRC*)	16.2	8	Iron import into cells
Lactotransferrin (*LTF*)	12.9	276	Iron import into cells
Haptoglobin (*HP*)	12.7	298	Binding protein - restrict free iron availability
Ferritin (*FTH1*)	12.5	580	Iron storage; ferroxidase activity.
SLC11A1	12.4	644	Iron export from phagolysosomes
Heme oxygenase 1 (*HMOX1*)	12.4	984	Intracellular heme catabolism.
Calprotectin (*CP*)	12.4	1025	Binding protein - restrict free iron availability
Hemopexin (*HPX*)	12.3	1637	Binding protein - restrict free iron availability
Hepcidin (*HAMP*)	12.2	1972	Inhibits iron absorption and export of intracellular iron.
Transferrin (*TF*)	9.4	3516	Iron import into cells
Hemoglobin. subunit beta (*HBB*)	9.3	3544	Binding protein - restrict free iron availability

References

1 Peetermans, M. *et al.* Von Willebrand factor and ADAMTS13 impact on the outcome of Staphylococcus aureus sepsis. *J Thromb Haemost* **18**, 722-731, doi:10.1111/jth.14686 (2020).

2 Gragnano, F. *et al.* The Role of von Willebrand Factor in Vascular Inflammation: From Pathogenesis to Targeted Therapy. *Mediators Inflamm* **2017**, 5620314, doi:10.1155/2017/5620314 (2017).

3 Peetermans, M. *et al.* Targeting Coagulase Activity in Staphylococcus aureus Bacteraemia: A Randomized Controlled Single-Centre Trial of Staphylothrombin Inhibition. *Thromb Haemost* **118**, 818-829, doi:10.1055/s-0038-1639586 (2018).

4 Vanassche, T. *et al.* Inhibition of staphylothrombin by dabigatran reduces Staphylococcus aureus virulence. *J Thromb Haemost* **9**, 2436-2446, doi:10.1111/j.1538-7836.2011.04529.x (2011).

5 Veloso, T. R. *et al.* Prophylaxis of experimental endocarditis with antiplatelet and antithrombin agents: a role for long-term prevention of infective endocarditis in humans? *J Infect Dis* **211**, 72-79, doi:10.1093/infdis/jiu426 (2015).

6 Hannachi, N. *et al.* Antiplatelet Agents Have a Distinct Efficacy on Platelet Aggregation Induced by Infectious Bacteria. *Front Pharmacol* **11**, 863, doi:10.3389/fphar.2020.00863 (2020).

7 Ulloa, E. R., Uchiyama, S., Gillespie, R., Nizet, V. & Sakoulas, G. Ticagrelor Increases Platelet-Mediated Staphylococcus aureus Killing, Resulting in Clearance of Bacteremia. *J Infect Dis* **224**, 1566-1569, doi:10.1093/infdis/jiab146 (2021).

8 Sun, J. *et al.* Repurposed drugs block toxin-driven platelet clearance by the hepatic Ashwell-Morell receptor to clear Staphylococcus aureus bacteremia. *Sci Transl Med* **13**, doi:10.1126/scitranslmed.abd6737 (2021).

9 Kaufmann, S. H. E., Dorhoi, A., Hotchkiss, R. S. & Bartenschlager, R. Host-directed therapies for bacterial and viral infections. *Nature Reviews Drug Discovery* **17**, 35-56, doi:10.1038/nrd.2017.162 (2018).

10 Watson, K. *et al.* Developing Novel Host-Based Therapies Targeting Microbicidal Responses in Macrophages and Neutrophils to Combat Bacterial Antimicrobial Resistance. *Front Immunol* **11**, 786, doi:10.3389/fimmu.2020.00786 (2020).

11 Cho, J. S. *et al.* IL-17 is essential for host defense against cutaneous Staphylococcus aureus infection in mice. *J Clin Invest* **120**, 1762-1773, doi:10.1172/jci40891 (2010).

12 Tal, M. C. *et al.* Upregulation of CD47 Is a Host Checkpoint Response to Pathogen Recognition. *mBio* **11**, doi:10.1128/mBio.01293-20 (2020).

13 Brown, D. Antibiotic resistance breakers: can repurposed drugs fill the antibiotic discovery void? *Nature Reviews Drug Discovery* **14**, 821-832, doi:10.1038/nrd4675 (2015).

14 Matussek, A. *et al.* Infection of human endothelial cells with Staphylococcus aureus induces transcription of genes encoding an innate immunity response. *Scand J Immunol* **61**, 536-544, doi:10.1111/j.1365-3083.2005.01597.x (2005).

15 Moreilhon, C. *et al.* Live Staphylococcus aureus and bacterial soluble factors induce different transcriptional responses in human airway cells. *Physiol Genomics* **20**, 244-255, doi:10.1152/physiolgenomics.00135.2004 (2005).

16 Ardura, M. I. *et al.* Enhanced monocyte response and decreased central memory T cells in children with invasive Staphylococcus aureus infections. *PLoS One* **4**, e5446, doi:10.1371/journal.pone.0005446 (2009).

17 Stark, L. *et al.* Staphylococcus aureus isolates from blood and anterior nares induce similar innate immune responses in endothelial cells. *Apmis* **117**, 814-824, doi:10.1111/j.1600-0463.2009.02535.x (2009).

18 Li, X. *et al.* Epithelial Cell Gene Expression Induced by Intracellular Staphylococcus aureus. *Int J Microbiol* **2009**, 753278, doi:10.1155/2009/753278 (2009).

19 Kobayashi, S. D. *et al.* Rapid neutrophil destruction following phagocytosis of Staphylococcus aureus. *J Innate Immun* **2**, 560-575, doi:10.1159/000317134 (2010).

20 Grundmeier, M. *et al.* Staphylococcal strains vary greatly in their ability to induce an inflammatory response in endothelial cells. *J Infect Dis* **201**, 871-880, doi:10.1086/651023 (2010).

21 Tchatalbachev, S., Ghai, R., Hossain, H. & Chakraborty, T. Gram-positive pathogenic bacteria induce a common early response in human monocytes. *BMC Microbiol* **10**, 275, doi:10.1186/1471-2180-10-275 (2010).

22 Ahn, S. H. *et al.* Two genes on A/J chromosome 18 are associated with susceptibility to Staphylococcus aureus infection by combined microarray and QTL analyses. *PLoS Pathog* **6**, e1001088, doi:10.1371/journal.ppat.1001088 (2010).

23 Banchereau, R. *et al.* Host immune transcriptional profiles reflect the variability in clinical disease manifestations in patients with Staphylococcus aureus infections. *PLoS One* **7**, e34390, doi:10.1371/journal.pone.0034390 (2012).

24 Lewandowska-Sabat, A. M. *et al.* The early phase transcriptome of bovine monocyte-derived macrophages infected with Staphylococcus aureus in vitro. *BMC Genomics* **14**, 891, doi:10.1186/1471-2164-14-891 (2013).

25 Nelson, C. L. *et al.* A genome-wide association study of variants associated with acquisition of Staphylococcus aureus bacteremia in a healthcare setting. *BMC Infect Dis* **14**, 83, doi:10.1186/1471-2334-14-83 (2014).

26 Ye, Z. *et al.* Genome wide association study of SNP-, gene-, and pathway-based approaches to identify genes influencing susceptibility to Staphylococcus aureus infections. *Front Genet* **5**, 125, doi:10.3389/fgene.2014.00125 (2014).

27 Gaviria-Agudelo, C., Carter, K., Tareen, N., Pascual, V. & Copley, L. A. Gene expression analysis of children with acute hematogenous osteomyelitis caused by Methicillin-resistant Staphylococcus aureus: correlation with clinical severity of illness. *PLoS One* **9**, e103523, doi:10.1371/journal.pone.0103523 (2014).

28 Yan, Q. *et al.* Dusp3 and Psme3 are associated with murine susceptibility to Staphylococcus aureus infection and human sepsis. *PLoS Pathog* **10**, e1004149, doi:10.1371/journal.ppat.1004149 (2014).

29 Brown, E. L. *et al.* Genome-Wide Association Study of Staphylococcus aureus Carriage in a Community-Based Sample of Mexican-Americans in Starr County, Texas. *PLoS One* **10**, e0142130, doi:10.1371/journal.pone.0142130 (2015).

30 Brady, R. A., Bruno, V. M. & Burns, D. L. RNA-Seq Analysis of the Host Response to Staphylococcus aureus Skin and Soft Tissue Infection in a Mouse Model. *PLoS One* **10**, e0124877, doi:10.1371/journal.pone.0124877 (2015).

31 DeLorenze, G. N. *et al.* Polymorphisms in HLA Class II Genes Are Associated With Susceptibility to Staphylococcus aureus Infection in a White Population. *J Infect Dis* **213**, 816-823, doi:10.1093/infdis/jiv483 (2016).

32 Yan, Q. *et al.* Candidate genes on murine chromosome 8 are associated with susceptibility to Staphylococcus aureus infection in mice and are involved with Staphylococcus aureus septicemia in humans. *PLoS One* **12**, e0179033, doi:10.1371/journal.pone.0179033 (2017).

33 Thänert, R., Goldmann, O., Beineke, A. & Medina, E. Host-inherent variability influences the transcriptional response of Staphylococcus aureus during in vivo infection. *Nat Commun* **8**, 14268, doi:10.1038/ncomms14268 (2017).

34 Lei, M. G., Gupta, R. K. & Lee, C. Y. Proteomics of Staphylococcus aureus biofilm matrix in a rat model of orthopedic implant-associated infection. *PLoS One* **12**, e0187981, doi:10.1371/journal.pone.0187981 (2017).

35 Moreau, K. *et al.* Human Genetic Susceptibility to Native Valve Staphylococcus aureus Endocarditis in Patients With S. aureus Bacteremia: Genome-Wide Association Study. *Front Microbiol* **9**, 640, doi:10.3389/fmicb.2018.00640 (2018).

36 Scott, W. K. *et al.* Human genetic variation in GLS2 is associated with development of complicated Staphylococcus aureus bacteremia. *PLoS Genet* **14**, e1007667, doi:10.1371/journal.pgen.1007667 (2018).

37 Chen, P. *et al.* Differentially Expressed Genes in Osteomyelitis Induced by Staphylococcus aureus Infection. *Front Microbiol* **9**, 1093, doi:10.3389/fmicb.2018.01093 (2018).

38 Sun, A. *et al.* Essential genes of the macrophage response to Staphylococcus aureus exposure. *Cell Mol Biol Lett* **23**, 25, doi:10.1186/s11658-018-0090-4 (2018).

39 Palma Medina, L. M. *et al.* Metabolic Cross-talk Between Human Bronchial Epithelial Cells and Internalized Staphylococcus aureus as a Driver for Infection. *Mol Cell Proteomics* **18**, 892-908, doi:10.1074/mcp.RA118.001138 (2019).

40 Bravo-Santano, N. *et al.* Identification of novel targets for host-directed therapeutics against intracellular Staphylococcus aureus. *Sci Rep* **9**, 15435, doi:10.1038/s41598-019-51894-3 (2019).

41 Toledo, A. G. *et al.* Proteomic atlas of organ vasculopathies triggered by Staphylococcus aureus sepsis. *Nat Commun* **10**, 4656, doi:10.1038/s41467-019-12672-x (2019).

42 Jacquet, R. *et al.* Dual Gene Expression Analysis Identifies Factors Associated with Staphylococcus aureus Virulence in Diabetic Mice. *Infect Immun* **87**, doi:10.1128/iai.00163-19 (2019).

43 Stelzner, K. *et al.* Intracellular Staphylococcus aureus Perturbs the Host Cell Ca(2+) Homeostasis To Promote Cell Death. *mBio* **11**, doi:10.1128/mBio.02250-20 (2020).

44 Michalik, S. *et al.* Early-Stage Staphylococcus aureus Bloodstream Infection Causes Changes in the Concentrations of Lipoproteins and Acute-Phase Proteins and Is Associated with Low Antibody Titers against Bacterial Virulence Factors. *mSystems* **5**, doi:10.1128/mSystems.00632-19 (2020).
45 Wozniak, J. M. *et al.* Mortality Risk Profiling of Staphylococcus aureus Bacteremia by Multi-omic Serum Analysis Reveals Early Predictive and Pathogenic Signatures. *Cell* **182**, 1311-1327.e1314, doi:10.1016/j.cell.2020.07.040 (2020).
46 Chang, Y. L. *et al.* Human DNA methylation signatures differentiate persistent from resolving MRSA bacteremia. *Proc Natl Acad Sci U S A* **118**, doi:10.1073/pnas.2000663118 (2021).
47 Xiao, S. J., Zhou, Y. F., Jia, H., Wu, Q. & Pan, D. F. Identification of the pivotal differentially expressed genes and pathways involved in Staphylococcus aureus-induced infective endocarditis by using bioinformatics analysis. *Eur Rev Med Pharmacol Sci* **25**, 487-497, doi:10.26355/eurrev_202101_24420 (2021).
48 Lin, Y. *et al.* mRNA Transcriptome Analysis of Bone in a Mouse Model of Implant-Associated Staphylococcus aureus Osteomyelitis. *Infect Immun* **89**, doi:10.1128/iai.00814-20 (2021).
49 Lindner, B. *et al.* A genome-wide CRISPR/Cas9 screen to identify phagocytosis modulators in monocytic THP-1 cells. *Sci Rep* **11**, 12973, doi:10.1038/s41598-021-92332-7 (2021).
50 Zhang, J. *et al.* Gene Expression Profile Analyses of the Skin Response of Balb/c-Nu Mice Model Injected by Staphylococcus aureus. *Clin Cosmet Investig Dermatol* **15**, 217-235, doi:10.2147/ccid.S348961 (2022).
51 Chen, Y. *et al.* Bone protein analysis via label-free quantitative proteomics in patients with periprosthetic joint infection. *J Proteomics* **252**, 104448, doi:10.1016/j.jprot.2021.104448 (2022).
52 Garcia-Moreno, M. *et al.* Osteocytes Serve as a Reservoir for Intracellular Persisting Staphylococcus aureus Due to the Lack of Defense Mechanisms. *Front Microbiol* **13**, 937466, doi:10.3389/fmicb.2022.937466 (2022).
53 Nicolas, A. *et al.* Transcriptome Architecture of Osteoblastic Cells Infected With Staphylococcus aureus Reveals Strong Inflammatory Responses and Signatures of Metabolic and Epigenetic Dysregulation. *Front Cell Infect Microbiol* **12**, 854242, doi:10.3389/fcimb.2022.854242 (2022).
54 Goldmann, O. *et al.* Cytosolic Sensing of Intracellular Staphylococcus aureus by Mast Cells Elicits a Type I IFN Response That Enhances Cell-Autonomous Immunity. *J Immunol* **208**, 1675-1685, doi:10.4049/jimmunol.2100622 (2022).
55 Dietrich, O. *et al.* Dysregulated Immunometabolism Is Associated with the Generation of Myeloid-Derived Suppressor Cells in Staphylococcus aureus Chronic Infection. *J Innate Immun* **14**, 257-274, doi:10.1159/000519306 (2022).
56 Müller, M. M. *et al.* Staphylococcus aureus induces tolerance in human monocytes accompanied with expression changes of cell surface markers. *Front Immunol* **14**, 1046374, doi:10.3389/fimmu.2023.1046374 (2023).
57 Sirichoat, A., Kaewseekhao, B., Nithichanon, A., Roytrakul, S. & Faksri, K. Proteomic Profiles and Protein Network Analysis of Primary Human Leukocytes Revealed Possible Clearance Biomarkers for Staphylococcus aureus Infection. *Curr Microbiol* **80**, 335, doi:10.1007/s00284-023-03450-6 (2023).
58 Fisher, C. R. *et al.* Mass spectrometry-based proteomic profiling of sonicate fluid differentiates Staphylococcus aureus periprosthetic joint infection from non-infectious failure: A pilot study. *Proteomics Clin Appl* **17**, e2200071, doi:10.1002/prca.202200071 (2023).
59 Thaden, J. T. *et al.* Use of Transcriptional Signatures to Differentiate Pathogen-Specific and Treatment-Specific Host Responses in Patients With Bacterial Bloodstream Infections. *J Infect Dis* **229**, 1535-1545, doi:10.1093/infdis/jiad498 (2024).
60 Zhao, Y. *et al.* Time-Course Transcriptome Analysis of the Lungs of Mice Challenged with Aerosols of Methicillin-Resistant Staphylococcus aureus USA300 Clone Reveals Inflammatory Balance. *Biomolecules* **13**, doi:10.3390/biom13020347 (2023).
